# NRF2 Knockdown Resensitizes 5-Fluorouracil-Resistant Pancreatic Cancer Cells by Suppressing HO-1 and ABCG2 Expression

**DOI:** 10.3390/ijms21134646

**Published:** 2020-06-30

**Authors:** Eui Joo Kim, Yoon Jae Kim, Hye In Lee, Seok-Hoo Jeong, Hyo Jung Nam, Jae Hee Cho

**Affiliations:** 1 Division of Gastroenterology, Department of Internal Medicine, Gil Medical Center, Gachon University College of Medicine, Incheon 21565, Korea; imejkim21@gmail.com (E.J.K.); yoonmed@gachon.ac.kr (Y.J.K.); wnsgpdls@hanmail.net (H.I.L.); butterfly_77@naver.com (H.J.N.); 2 Division of Gastroenterology, Department of Internal Medicine, Catholic Kwandong University International St. Mary’s Hospital, Incheon 22711, Korea; ssukoo@naver.com; 3 Division of Gastroenterology, Department of Internal Medicine, Gangnam Severance Hospital, Yonsei University College of Medicine, Seoul 06273, Korea

**Keywords:** pancreatic cancer, 5-Fluorouracil, chemoresistance, NF-E2-related factor 2

## Abstract

Chemoresistance is a leading cause of morbidity and mortality in patients with pancreatic cancer and remains an obstacle to successful treatment. The antioxidant transcription factor nuclear factor (erythroid-derived 2)-related factor 2 (NRF2), which plays important roles in tumor angiogenesis and invasiveness, is upregulated in pancreatic ductal adenocarcinoma (PDAC), where it correlates with poor survival. Here, we investigated the role of NRF2 in two 5-Fluourouracil-resistant (5-FUR) PDAC cell lines: BxPC-3 and CFPAC-1. Levels of NRF2 and antioxidants, such as heme oxygenase 1 (HO-1), NAD(P)H quinone dehydrogenase 1 (NQO1), and superoxide dismutase 2 (SOD2), were higher in the chemoresistant cells than in their chemosensitive counterparts. Expression of epithelial mesenchymal transition (EMT) markers, stemness markers, including Nanog, Oct4, and CD133, and that of the drug transporter ATP binding cassette, subfamily G, member A2 (ABCG2) was also upregulated in 5-FUR PDAC cells. NRF2 knockdown reversed 5-FU resistance of PDAC cells via suppression of ABCG2 and HO-1. In summary, these data indicate that NRF2 is a potential target for resensitizing 5-FUR PDAC cells to 5-FU to improve treatment outcomes in patients with pancreatic cancer.

## 1. Introduction

Pancreatic ductal adenocarcinoma (PDAC) is one of the most aggressive malignancies and a leading cause of cancer-related mortality. Surgical resection is the only curative therapeutic option; however, only 15–20% of patients have resectable tumors at initial diagnosis [1,2]. Most patients with locally advanced or metastatic PDAC require systemic chemotherapy and are usually treated with a combination of fluorouracil, leucovorin, irinotecan, and oxaliplatin (FOLFIRINOX), which should be considered first-line treatment in carriers of the breast cancer gene (BRCA) or BRCA-like mutations [3,4,5]. 5-Fluorouracil (5-FU), a component of FOLFIRINOX, is a long-established anticancer drug that was traditionally used to treat PDAC. However, acquisition of drug resistance and cytotoxicity at high concentrations have rendered 5-FU therapy inefficient. Chemoresistance is a major hindrance to successful treatment of many tumors. Thus, it is crucial to elucidate the mechanisms of chemoresistance and develop therapeutic strategies to resensitize cancer cells to chemotherapy [6,7].

Nuclear factor (erythroid-derived 2)-related factor 2 (NRF2) is a transcription factor that plays a critical role in cellular defense against oxidative stress and xenobiotics through induction of antioxidants, such as heme oxygenase-1 (HO-1), NAD(P)H quinone dehydrogenase 1 (NQO1), and ATP-binding cassette (ABC) transporters [8,9]. Accumulating evidence shows that NRF2 is overactivated in cancer cells, and that aberrant NRF2 signaling creates an environment that promotes tumorigenesis, invasiveness, and chemoresistance [9,10,11,12,13,14]. Nuclear NRF2 expression is related to poor survival in human PDAC [15].

The present study aimed to explore the relationship between NRF2 and chemoresistance in two 5-FU-resistant (5-FUR) PDAC cell lines, BxPC-3 and CFPAC-1, and elucidate the underlying molecular mechanisms.

## 2. Results

### 2.1. NRF2 Is Overexpressed in 5-FU-Resistant Human Pancreatic Cancer Cells

**Figure 1:**
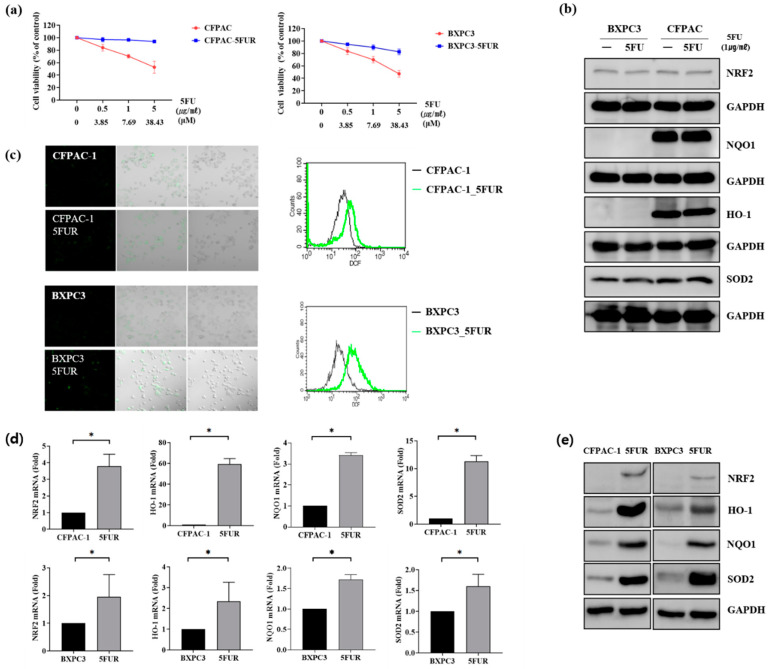
Establishment of 5-Fluourouracil-resistant (5-FUR) human pancreatic cancer cells (CPFAC-1 and BxPC-3). (**a**) Viability of human pancreatic ductal adenocarcinoma (PDAC) cells and their 5-FUR counterparts following treatment with increasing doses of 5-Fluourouracil (5-FU) for 48 h, as measured by the MTT assay; (**b**) Western blotting showed the expression of nuclear factor (erythroid-derived 2)-related factor 2 (NRF2), NAD(P)H quinone dehydrogenase 1 (NQO1), heme oxygenase-1 (HO-1), and superoxide dismutase 2 (SOD2) was not changed in control cell lines with 5-FU treatment (1 µg/mL) for 24 h. (**c**) Intracellular reactive oxygen species (ROS) levels were higher in 5-FUR PDAC cells than in control PDAC cells, based on 2′,7′-dichlorofluorescein diacetate (H_2_DCFDA) staining; (**d**) RT-qPCR showed that the mRNA expression levels of NRF2 (*n* > 5), HO-1 (*n* = 3), NQO1 (*n* = 3), and SOD2 (*n* = 3) were higher in 5-FUR PDAC cells than in parental cells; (**e**) Western blotting confirmed that expression of NRF2, HO-1, NQO1, and SOD2 was upregulated in 5-FUR PDAC cells compared with parental cells. * *p* < 0.05.

5-FUR BxPC-3 and 5-FUR CPFAC-1 cell lines were established to examine the mechanism of 5-FU resistance in pancreatic cancer. The cells were exposed to increasing doses of 5-FU, and the half-maximal inhibitory concentration (IC50) values were determined using the 3-(4,5-dimethylthiazol-2-yl)-2,5-diphenyltetrazolium bromide (MTT) assay. 5-FU had a higher IC50 value in 5-FUR PDAC cells than in parental PDAC cells (Figure 1a). Moreover, the levels of intracellular reactive oxygen species (ROS), NRF2, and antioxidants, such as HO-1, NQO1, and superoxide dismutase 2 (SOD2), were elevated in 5-FUR PDAC cells compared with control PDAC cells (Figure 1b–e).

### 2.2. EMT Markers, Stemness Markers, and ATP Binding Cassette, Subfamily G, Member A2 (ABCG-2) Are Upregulated in 5-FU-Resistant Human Pancreatic Cancer Cells

**Figure 2:**
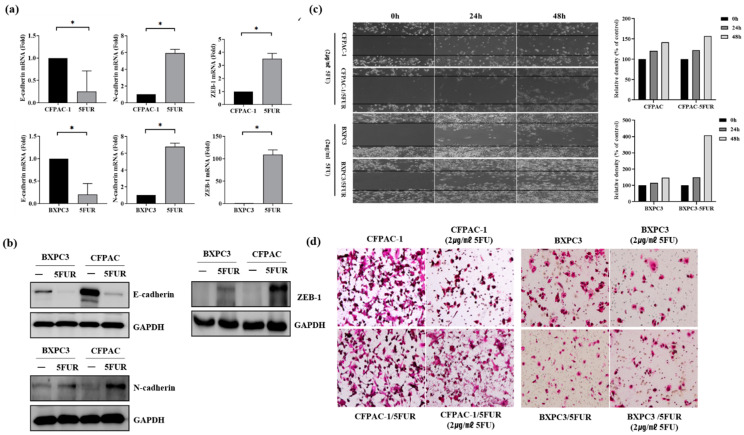
Epithelial mesenchymal transition (EMT) marker expression in 5-FUR human pancreatic cancer cells (CPFAC-1 and BxPC-3). (**a**) E-cadherin (*n* = 3) was downregulated, while N-cadherin (*n* = 3) was upregulated in 5-FUR PDAC cells, as shown by RT-qPCR; (**b**) Western blotting confirmed overexpression of N-cadherin and Zinc finger E-box-binding homeobox 1 (ZEB-1) in 5-FUR PDAC cells; (**c**,**d**) migration and invasion assay showed that 5-FUR pancreatic cancer cells possessed higher migration and invasion ability than did control cells (CPFAC-1 and BxPC-3). * *p* < 0.05.

**Figure 3:**
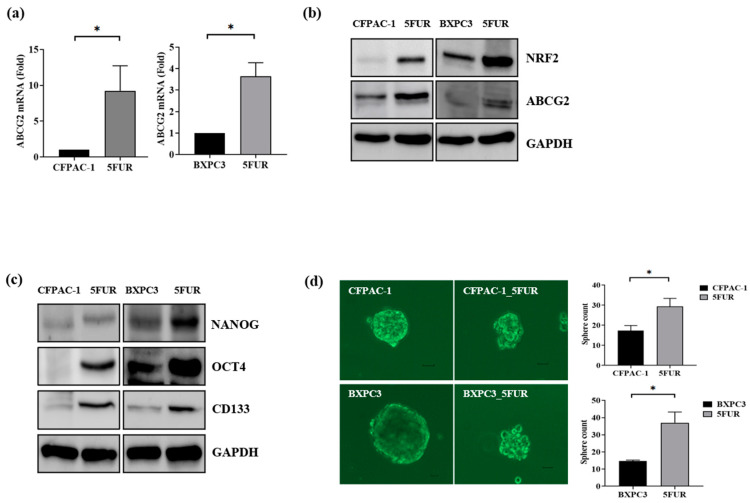
Stem cell characteristics of 5-FUR human pancreatic cancer cells (CPFAC-1 and BxPC-3). (**a**,**b**) ABCG-2 (*n* = 3) was upregulated in 5-FUR PDAC cells, as shown by RT-qPCT and Western blotting; (**c**) Nanog, Oct4, and CD133 were overexpressed in 5-FUR PDAC cells; (**d**) 5-FUR PDAC cells had higher sphere-forming ability than control PDAC cells. * *p* < 0.05.

The relationship between NRF2 and epithelial mesenchymal transition (EMT) was assessed using RT-qPCR and Western blotting. E-cadherin was downregulated, while N-cadherin and Zinc finger E-box-binding homeobox 1 (ZEB1) were upregulated in 5-FUR PDAC cells (Figure 2a,b). Further, 5-FUR PDAC cells had a higher migration rate than parental cells (Figure 2c,d). The multidrug-resistant efflux pump of the ABC, subfamily G, member A2 (ABCG-2), and stemness markers, including Oct4, Nanog, and CD133, were overexpressed in 5-FUR PDAC cells (Figure 3a–c). Additionally, 5-FUR PDAC cells had a higher sphere-forming ability than control cells (Figure 3d).

### 2.3. Silencing of NRF2 Resensitizes 5-FU-Resistant Human Pancreatic Cancer Cells to 5-FU by Suppressing HO-1 and ABCG2

**Figure 4:**
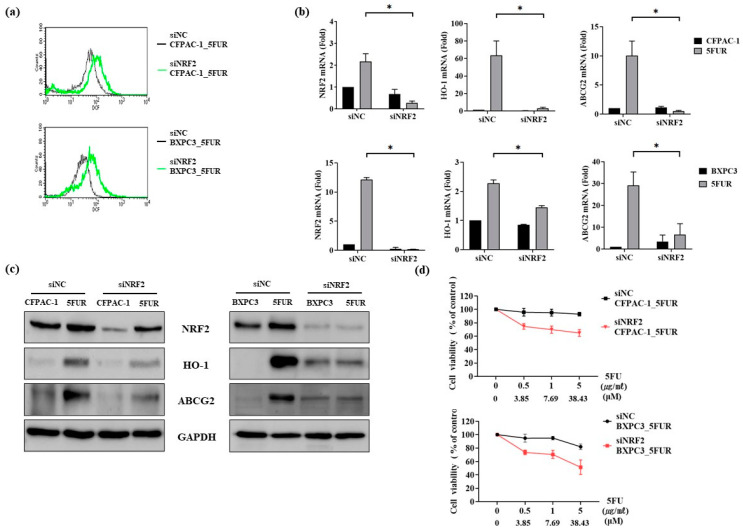
Effects of NRF2 knockdown on 5-FUR pancreatic cancer cells (CPFAC-1 and BxPC-3). (**a**) DCFDA staining revealed that ROS levels were higher in siNRF2 5-FUR PDAC cells than in the siNC 5-FUR PDAC cells; (**b**) expression of NRF2 (*n* = 5), HO-1 (*n* = 3), NQO-1 (*n* = 3), and ABCG-2 (*n* = 3) was downregulated in siNRF2 5-FUR PDAC cells, as determined by RT-qPCR; (**c**) Western blotting confirmed that levels of ABCG-2, HO-1, and N-cadherin were decreased, while those of E-cadherin were increased in siNRF2 5-FUR PDAC cells; (**d**) MTT assay results revealed that the antitumor effect of 5-FU was significantly more potent in siNRF2 5-FUR PDAC cells than in the negative control (NC) cells. * *p* < 0.05.

To gain mechanistic insights into the role of NRF2 in 5-FU resistance of PDAC cell lines, we knocked down NRF2 expression using a small interfering RNA (siRNA)-mediated approach. NRF2-silenced 5-FUR PDAC cells showed decreased expression of NRF2 and its downstream target HO-1 and had lower levels of ROS than the negative control (NC) 5-FUR PDAC cells (Figure 4a).

Regarding EMT markers, levels of E-cadherin were higher, while those of N-cadherin were lower in siNRF2 5-FUR PDAC cells than in the NC cells. The mRNA and protein expression of ABCG2 was also decreased in NRF2-silenced cells (Figure 4b,c). Both CPFAC-1 and BxPC-3 siNRF2 5-FUR cells were more vulnerable to 5-FU than the corresponding NC 5-FUR cells (*p* < 0.05, Figure 4d).

## 3. Discussion

FOLFIRINOX is recommended as first-line chemotherapy for PDAC; thus, overcoming chemoresistance to its components, including 5-FU, is the most important aspect of the management of this lethal disease. 5-FU acts as an antimetabolite and irreversibly inhibits thymidylate synthase, interfering with DNA and RNA synthesis and consequently inhibits cell growth and induces apoptosis [6]. Here, we established 5-FUR BXPC-3 and CFPAC1 cells and investigated the molecular mechanism of their chemoresistance to help improve therapeutic outcomes of PDAC treatment. Our results revealed that compared with parental PDAC cells, their 5-FUR counterparts displayed higher levels of NRF2 and its downstream antioxidants, including HO-1, NQO1, and SOD2. Furthermore, 5-FUR PDAC cells had greater sphere-forming ability and higher levels of EMT markers and the multidrug resistance-associated protein transporter ABCG2 than did control PDAC cells.

Because NRF2 was overexpressed in both 5-FUR BxPC-3 and 5-FUR CFPAC-1 cells, we focused on the relationship between NRF2 and 5-FU chemoresistance. NRF2 is a key regulator of the antioxidant response in normal cells. In cancer cells, prolonged NRF2 overexpression induced by excessive production of ROS promotes cancer progression, invasion, metastasis, and chemoresistance. Furthermore, high levels of NRF2 are associated with high metastatic potential driven by EMT. Short hairpin RNA (shRNA)-mediated knockdown of NRF2 in esophageal squamous cell carcinoma suppressed EMT, decreasing migration and invasion of cancer cells [16,17]. Thus, NRF2 overexpression is a poor prognostic factor in patients with PDAC, as well as in other cancers [11,12,13,18,19]. The NRF2/HO-1 axis is a major signaling pathway downstream of NRF2 [19]. HO-1 is the inducible form of heme oxygenase and acts as the first rate-limiting enzyme in the process of degradation of free heme, carbon monoxide, and ferritin [20,21,22]. HO-1 is involved in the maintenance of cellular homeostasis and adaptation to cellular stress, as well as being implicated in cancer development, aggressiveness, and poor outcomes in gallbladder cancers, non-small cell lung cancer, cervical cancer, hepatoma, esophageal squamous cell carcinoma, and multiple myeloma [17,23,24,25].

5-FU-resistant cells appear to have cancer stem cell-like properties and express the drug transporter ABCG-2. In the present study, 5-FUR PDAC cells upregulated their expression of cancer stem cell markers Nanog, Oct4, and CD133, and ABCG2 and displayed higher sphere-formation ability than did control PDAC cells. NRF2 knockdown resensitized 5-FUR PDAC cells to 5-FU by suppressing NRF2/HO-1 signaling and ABCG2 expression. These observations support the idea that cancer stemness and ABCG-2 expression are responsible for 5-FU resistance in PDAC. The relationship between NRF2 and chemoresistance has been reported previously. Recent studies demonstrate that NRF2 plays a crucial role in drug resistance of cancer stem cells [9,26] and is essential for preserving tumorigenicity and self-renewal in glioma stem cells and breast cancer stem cells [27,28]. Moon et al. showed that high expression of NRF2 confers resistance to chemotherapeutic drugs, including etoposide, cisplatin, and doxorubicin [18]. The NRF2/HO-1 axis is reportedly activated in neuroblastoma after bortezomib treatment [29], in cisplatin-treated ovarian carcinoma cells [30], and in doxorubicin-resistant breast cancer cells [31]. NRF2 knockdown in lung cancer cells depleted their ABCG2 levels and sensitized them to the chemotherapeutic drugs mitoxantrone and topotecan [32]. Taken together, our data indicate that NRF2 is a major regulator of 5-FU resistance in PDAC cells. Suppression of NRF2 decreases HO-1 and ABCG2 expression, which may increase drug sensitivity, making NRF2 a novel target for the treatment of PDAC.

Several limitations of our study ought to be noted. First, it was recently shown that oncogenic KRAS-induced NRF2 upregulates glutaminolysis, which promotes chemoresistance in PDAC [14,33]; however, we were unable to explore the role of NRF2 in cancer metabolism in the present study. Second, we used PDAC cell lines rather than patient-derived pancreatic cancer cells and did not confirm the relationship between NRF2 and target proteins, such as ABCG2, in vivo. Further studies are warranted to investigate the connection between NRF2, drug transporters, and cell signaling pathways in cancer cells.

In conclusion, our study highlights the mechanistic features of NRF2-mediated 5-FU resistance in PDAC cells and provides a rationale for targeting the NRF2 regulatory pathway to confer therapeutic opportunities for PDAC that could be translated into clinical trials.

## 4. Materials and Methods

### 4.1. Reagents and Materials

The 5-FU (Sigma-Aldrich Inc., St. Louis, MO, USA) was diluted in dimethyl sulfoxide and stored at −20 °C. Primary antibodies against the following proteins were used: NRF2 (sc-365949; Santa Cruz Biotechnology Inc., CA, USA), HO-1 (sc-136960; Santa Cruz Biotechnology Inc., CA, USA), SOD2 (LF-PA0021; Ab Frontier, Seoul, Korea), NQO-1 (#3187; Cell Signaling Technology, MA, USA), P-P38 (#4511; Cell Signaling Technology, MA, USA), P38 (#8690; Cell Signaling Technology, MA, USA), E-cadherin (#3195; Cell Signaling Technology, MA, USA), N-cadherin (#13116; Cell Signaling Technology, MA, USA), ZEB-1 (#3396; Cell Signaling Technology, MA, USA), ABCG2 (sc-377176; Santa Cruz Biotechnology Inc., CA, USA), Nanog (#3580; Cell Signaling Technology, MA, USA), Oct4 (ab18976; Abcam, MA, USA), CD133 (MAB4310; Millipore Billerica, MA, USA), and glyceraldehyde 3-phosphate

Dehydrogenase (GAPDH) (sc-47724; Santa Cruz Biotechnology Inc., CA, USA). Horseradish peroxidases (HRP)-conjugated anti-rabbit and anti-mouse IgG antibodies were obtained from Millipore (AP132P, AP124P; Billerica, MA, USA).

### 4.2. Cell Lines and Cell Culture Conditions

Human pancreatic cancer cell lines CFPAC-1 and BxPC-3 were obtained from the American Type Culture Collection. The cells were cultured in Iscove’s Modified Dulbecco’s Medium (IMDM) and Roswell Park Memorial Institute (RPMI) medium supplemented with 10% fetal bovine serum (FBS; Gibco, Grand Island, NY, USA) and 1% penicillin–streptomycin, and maintained at 37 °C in a humidified incubator with 5% CO_2_. Cells resistant to 5-FU were selected as previously reported [34]. The cell line was incubated with serial treatment of escalating dosages of 5-FU from 0.5 up to 2 µg/mL (0.5 µg/mL for three passages, 1.0 µg/mL for three passages, and 2 µg/mL for three passages) for three weeks. To ensure maintenance of chemoresistance, 5-FUR CFPAC-1 and 5-FUR BxPC-3 cells were grown in the presence of 2 µg/mL 5-FU.

### 4.3. Sphere Culture

The cells were maintained in Dulbecco’s Modified Eagle’s Medium (DMEM)–F-12 containing B-27 supplement (GIBCO., Invitrogen, Carlsbad, CA, USA), N-2 supplement (GIBCO., Invitrogen, Carlsbad, CA, USA), 1% penicillin–streptomycin, 50 ng/mL basic fibroblast growth factor (bFGF, R&D System, Minneapolis, MN, USA), and 50 ng/mL epidermal growth factor (EGF, R&D System, Minneapolis, MN, USA), and cultured on an ultra-low attachment plate at 37 °C under 5% CO_2_.

### 4.4. Cell Viability

The cells (1 × 10^4^/well) were seeded in a 96-well plate. After 24 h of incubation, increasing concentrations of 5-FU were added. Subsequently, water soluble tetrazolium salt (WST) solution (20 µL; EN-CYTOX, DOGEN) was added to each well and the cells were incubated at 37 °C under 5% CO_2_ for 3 h. Absorbance was measured at 450 nm with a microplate reader (E-MAX, Molecular Devices).

### 4.5. Quantitative Reverse Transcription Polymerase Chain Reaction (RT-qPCR)

The abundance of NRF2 and HO-1 transcripts was measured by RT-qPCR. In brief, purified total RNA was converted into first-strand cDNA using RNAiso Plus (Takara Bio Inc., Shiga, Japan) and PrimeScript^TM^ RT reagent Kit (Takara Bio Inc., Shiga, Japan), respectively. First-strand cDNA of the target gene was then amplified using specific primers with the TaKaRa SYBR Premix Ex Taq II (TaKaRa, Kusatsu, Japan) on an iCycler (Bio-Rad, Hercules, CA, USA).

The following primer pairs were used: human NRF2, 5′-GAG AGC CCA GCT TTC ATT GC-3′ and 5′-TTG GCT TCT GGA CTT GGA AC-3′; human GAPDH, 5′-AGG GCT GCT TTT AAC TCT GGT-3′ and 5′-CCC CAC TTG ATT TTG GAG GGA-3′; human HO-1, 5′-ATG ACA CCA AGG ACC AGA GC-3′ and 5′-GTG TAA GGA CCC ATC GGA GA-3′; human NQO-1, 5′-AAA GGA CCC TTC CGG AGT AA-3′ and 5′-CCA TCC TTC CAG GAT TTG AA-3′; human P38, 5′-AGA ACA ACA CTT CCG TGT GCT-3′ and 5′-TGC AGT GAG CGT GAT TGT G-3′; human E-cadherin, 5′-TGC CCA GAA AAT GAA AAA GG-3′ and 5′-GTG TAT GTG GCA ATG CGT TC-3′; human N-cadherin, 5′- TGG ATG GAC CTT ATG TTG CT-3′ and 5′-AAC ACC TGT CTT GGG ATC AA-3′; human SOD2, 5′-ATG TTG AGC CGG GCA GTG TG-3′ and 5′-GTG CAG CTG CAT GAT CTG CG-3′; human ZEB-1, 5′-GCA CCT GAA GAG GAC CAG AG-3′ and 5′-GTG TAA CTG CAC AGG GAG CA-3′; human ABCG2, 5′-CAC TGA GAT TTG GGC TGC TT-3′; and 5′-TGG AAG ACA TCT GGA GAG TTT TTA T-3′. Target gene expression was normalized to that of GAPDH. The relative gene expression was set to 1-fold for the untreated control, and the normalized fold change ratio was calculated using the 2^−ΔΔ*C*t^ method.

### 4.6. Transfection of Small Interfering RNAs

Small interfering RNAs (siRNAs) against NFE2L2 were purchased from Dharmacon (Thermo, Lafayette, CO, USA). The siRNAs were dissolved in RNase-free H_2_O, diluted, then transfected into cells using Lipofectamine RNA iMAXTM (Invitrogen, Carlsbad, CA, USA). Scrambled siRNA was used as a negative control.

### 4.7. Measurement of Reactive Oxygen Species (ROS)

The levels of intracellular ROS were measured using H_2_DCFDA dye (Invitrogen, Carlsbad, CA, USA). At the indicated times, the dye was added to the culture media to a final concentration of 50 μM. After 15 min of incubation, intracellular fluorescence was observed with an inverted fluorescence microscope (ZEISS, Land Baden- Württemberg, Germany). Flow cytometry was performed using a BD FACSCalibur flow cytometer (BD Biosciences, Durham, NC, USA).

### 4.8. Invasion Assay

CFPAC-1 and BxPC-3 cells were seeded at a density of 5 × 10^4^ cells/well in a 24-well chamber of a Matrigel-coated Transwell insert (Corning Incorporated, Corning, NY, USA). IMDM and RPMI media supplemented with 0.1% FBS were added to the upper chamber, while 10% FBS was added to the lower chamber, and the cells were incubated for 24 h. The next day, the Transwell inserts were removed, washed with PBS, fixed in methanol, and stained with hematoxylin and eosin. The number of invaded cells was counted using a light microscope (BX51, Olympus, Tokyo, Japan).

### 4.9. Migration Assay (Wound Healing Assay)

CFPAC-1 and BxPC-3 cells were cultured in 6-well dishes. After reaching 80% confluence, a scratch was made in each well using a 200 μL pipette tip. To remove cell debris generated by the scratch, each well was washed once with sterile phosphate-buffered saline (PBS) and the culture media were replaced subsequently. Cell migration was evaluated under an inverted microscope at 0, 24, and 48 h.

### 4.10. Sphere Formation Assay

The sphere formation assay was performed based on a previously described method. CFPAC-1 and BxPC-3 cells were isolated from cell culture media before the single cell suspension (5000 cells/well) was plated on an ultra-low attachment 6-well plate, and incubated for 14 days. At the end of incubation, the spheres were evaluated and counted under an inverted microscope.

### 4.11. Statistical Analysis

The results are expressed as mean ± standard deviation of more than three independent experiments. Statistical comparisons were performed using one-way analysis of variance (ANOVA) with Tukey’s post-hoc test. Differences with *p* < 0.05 were considered statistically significant. Graphs were prepared using GraphPad Prism software version 8.0 (GraphPad Software Inc., La Jolla, CA, USA).

## Abbreviations

NRF2	Nuclear factor erythroid-derived 2-related factor 2
PDAC	Pancreatic ductal adenocarcinoma
5-FU	5-Fluourouracil
5-FUR	5-Fluourouracil-resistant
HO-1	Heme oxygenase 1
NQO1	NAD(P)H Quinone Dehydrogenase 1
SOD2	Superoxide dismutase 2
EMT	Epithelial mesenchymal transition
ABC	ATP-binding cassette
IC50	Maximal half inhibitory concentration
ROS	Reactive oxygen species
ABCG-2	ATP binding cassette, subfamily G, member A2
NC	Negative control
shRNA	Short hairpin RNA
